# Identification of Differentially Expressed Gene after Femoral Fracture via Microarray Profiling

**DOI:** 10.1155/2014/208751

**Published:** 2014-07-08

**Authors:** Donggen Zhong

**Affiliations:** Department of Physical Education, Jiangxi University of Finance and Economics, Nanchang 330013, China

## Abstract

We aimed to investigate differentially expressed genes (DEGs) in different stages after femoral fracture based on rat models, providing the basis for the treatment of sport-related fractures. Gene expression data GSE3298 was downloaded from Gene Expression Omnibus (GEO), including 16 chips. All femoral fracture samples were classified into earlier fracture stage and later fracture stage. Total 87 DEGs simultaneously occurred in two stages, of which 4 genes showed opposite expression tendency. Out of the 4 genes, *Rest* and *Cst8* were hub nodes in protein-protein interaction (PPI) network. The GO (Gene Ontology) function enrichment analysis verified that nutrition supply related genes were enriched in the earlier stage and neuron growth related genes were enriched in the later stage. Calcium signaling pathway was the most significant pathway in earlier stage; in later stage, DEGs were enriched into 2 neurodevelopment-related pathways. Analysis of Pearson's correlation coefficient showed that a total of 3,300 genes were significantly associated with fracture time, none of which was overlapped with identified DEGs. This study suggested that *Rest* and *Cst8* might act as potential indicators for fracture healing. Calcium signaling pathway and neurodevelopment-related pathways might be deeply involved in bone healing after femoral fracture.

## 1. Introduction

As the 2008 Beijing Olympics were successfully held in Beijing, sports developed rapidly in China. More and more inhabitants, professional or amateur, take part in daily physical activities. However, improper movement may cause injury. The intense sports (like pugilism, football, and basketball) and hazardous sports (like motorcycle race, drift motion, and bungee jumping) are all high-risk sports. Collisions with the ground, objects, and other players are common, and unexpected dynamic force on limbs and joints can cause injury [1]. In human, the femur fracture is one of the most common injuries resulted from improper movement [2]. The femur is the only bone in the thigh with the formation of long, slender, and cylindrical bone and is capable of walking, running, or jumping [3]. The femoral fracture is involved in the femoral head, femoral neck, or the shaft of the femur, accounting for 1-2% of all fractures in children and adolescents [4, 5].

For a long time, femoral fractures have been treated by using traction and/or casting [6]. More recently, surgery has gained popularity [7]. However, femur fracture is still difficult to manage because of the multifocal fractures of the femur [8, 9]. Although numerous surgical operations have been described to manage this injury, evidence for which to choose is lacking and individual approach is strongly emphasized during the treatment of these injuries [9, 10]. It is necessary for us to study the differences of gene expression at different stages after femoral fracture, in the purpose of finding the indicator of fracture healing.

Rats grow rapidly to attain their adult size. At 4 weeks, femur growth is near its maximum rate. At the age of 10 weeks, the linear growth of femur has slowed due to mitosis and hypertrophy in the chondrocytes of the physis [11, 12]. Based on rat model, changes in mRNA gene expression of femoral heading have been reported [12, 13]. Briefly, in 4-week-old female Sprague-Dawley (SD) rats, at 0 (intact), 0.1, 0.4, 1, 2, 3, 4, and 6 weeks after fracture, mRNA gene expression in the femoral heading after unilateral midshaft femoral fracture was identified, including 8,002 genes, about half increasing and half decreasing. These upregulated genes were related to cartilage, blood vessels, osteoprotegerin, osteomodulin, and most ribosomal proteins. Meanwhile, downregulated genes were related to bone, growth promoting cytokines, G proteins, GTPase-mediated signal transduction factors, cytokine receptors, mitosis, integrin-linked kinase, and the cytoskeleton. The relevant microarray data were deposited in GEO (Gene Expression Omnibus) database (ID: GSE3298) [12, 13].

In this present study, based on the microarray data of GSE3298, 2 weeks after femoral fracture was chosen as a split point, and thus the earlier stage and later stage were grouped. We aimed to identify DEGs at different stages of femoral fracture healing by bioinformatics methods, in order to provide the basis for the treatment of sport-related fractures.

## 2. Materials and Methods

### 2.1. Microarray Data

The mRNA expression profiling data was obtained from the research of Meyer et al., which were displayed in GEO (http://www.ncbi.nlm.nih.gov/geo/) database (ID: GSE3298) [12]. Briefly, female SD rats, aged 4 weeks at surgery, were subjected to a unilateral, simple, transverse, and middiaphyseal femoral fracture and stabilized with an intramedullary rod. At 0 (intact), 0.1, 0.4, 1, 2, 3, 4, and 6 weeks after fracture, the femoral head with the proximal physis was collected from fractured and intact femora. The RNA was extracted, processed to biotin labeled cRNA, and hybridized to Affymetrix Rat 230 2.0 GeneChip microarrays. The full microarray data has been deposited in the NCBI GEO as series GSE3298.

### 2.2. Data Preprocess

The microarray data in CEL files were downloaded from GEO database, including 16 chips, converted into fluorescence intensity values and standardized via the robust multiarray average (RMA) method [14]. For genes corresponding to multiple probe sets that had a plurality of expression values, the expression values of those probe sets were summed.

### 2.3. Differentially Expressed Gene Analysis

Considering the different healing level in different periods after fracture, 2 weeks was set as the split point. Chips data were divided into 2 groups: earlier stage (0.1, 0.4, 1 and 2 weeks after fracture) and later stage (2, 3, 4, and 6 weeks after fracture). The LIMMA package in R language was used to identify DEGs between earlier stage and later stage [15]. The* P* value <0.05 and the |log⁡_2_FC| > 0.5 were used as the cut-off criterion.

### 2.4. Construction of Interaction Network

For genes differentially expressed in two stages, the STRING (Search Tool for the Retrieval of Interacting Genes) [16] database was used to analyze their interaction network. For genes with consistent expression in two stages, BisoGenet [17] software was performed to map these genes to STRING database or BOND database for interaction network analysis. The *P* value <0.05 was chosen as cut-off criterion.

### 2.5. Pathway Enrichment Analysis

For function analysis of DEGs, the DEGs of two stages were, respectively, inputted into DAVID (Database for Annotation, Visualization, and Integrated Discovery) [18, 19] for KEGG (Kyoto Encyclopedia of Genes and Genomes) [20] pathway and GO (Gene Ontology) [21] enrichment analysis. The count number larger than 5 and *P* value less than 0.01 were chosen as cut-off criterion.

### 2.6. Correlation Analysis

A Pearson correlation coefficient was calculated between expression level of every expressed gene after fracture and fracture time via cor.test in R language [22]. The *P* < 0.05 was chosen as cut-off criterion. Then, DAVID tool was used to identify function classification associated with these significant genes.

## 3. Results

### 3.1. Differentially Expressed Genes

After standardization, there were 31,042 probes corresponding to 30,641 genes. In earlier stage, total 1,004 DEGs had been identified, including 301 upregulated genes and 703 downregulated genes. In later stage, total 986 DEGs were obtained, including 446 upregulated genes and 540 downregulated genes. The most significant DEGs from two stages were displayed in Tables 1 and 2.

**Figure 1:**
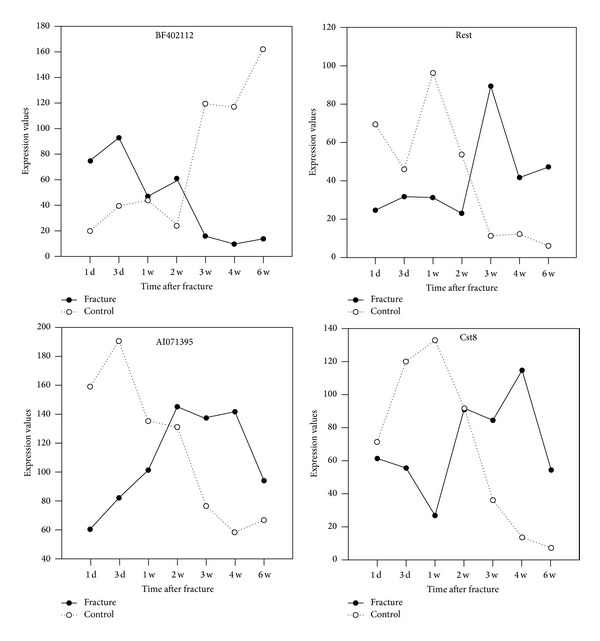
Differentially expressed genes showed contrary regulation tendency in earlier stage and later stage.

Among DEGs from two stages, 87 DEGs occurred in both earlier stage and later stage, including 26 upregulated genes, 57 downregulated genes, and 4 differentially regulated genes. Briefly, one DEG (GenBankAcc: BF402112) was upregulated in earlier stage and downregulated in later stage, and 3 DEGs (GenBankAcc: AF037203 (*Rest*), AI071395, and NM019258 (*Cst8*)) were downregulated in earlier stage and upregulated in later stage (Figure 1).

### 3.2. Interaction Network of DEGs

**Figure 2:**
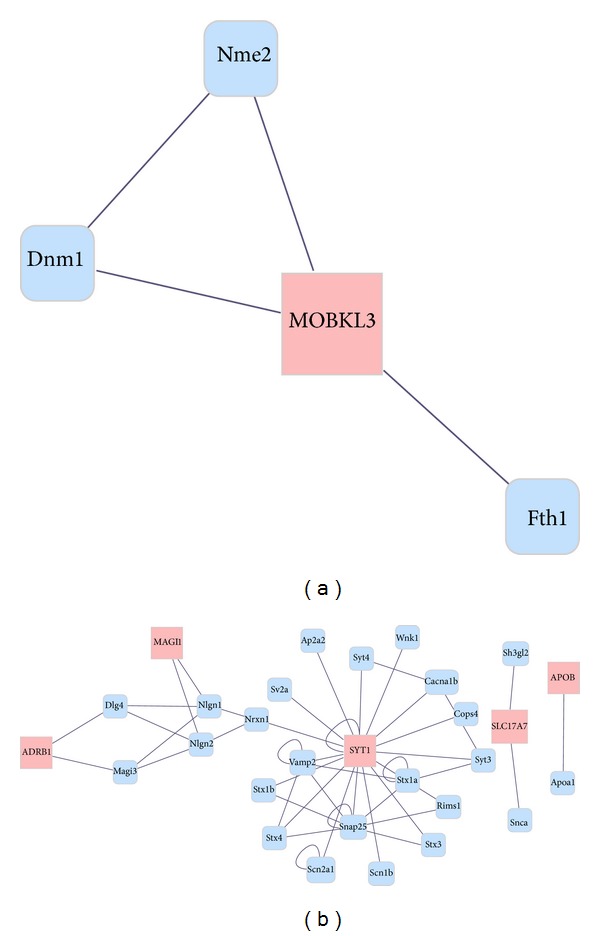
The interaction network of the obtained 87 DEGs. (a) The interaction network of 26 upregulated DEGs. (b) The interaction network of 57 downregulated DEGs. Red boxes: DEGs; blue boxes: reported gene in rats.

The obtained 87 DEGs were mapped to STRING in order to construct the interaction network. Among the 26 upregulated DEGs, only* MOBKL3 *was the hub node that interacted with other genes (Figure 2(a)). Meanwhile, among the 57 downregulated DEGs, 5 DEGs showed interaction with other genes in rats (Figure 2(b)), such as syt and stx families.

**Figure 3:**
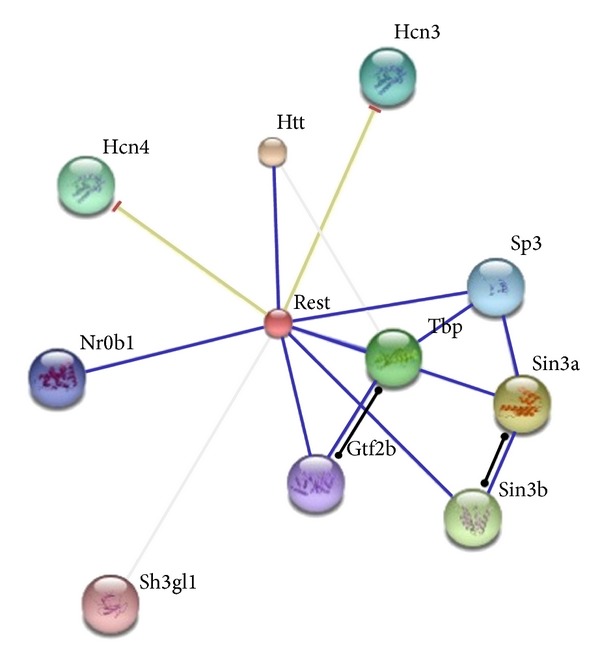
The PPI network of* Rest *gene.

**Figure 4:**
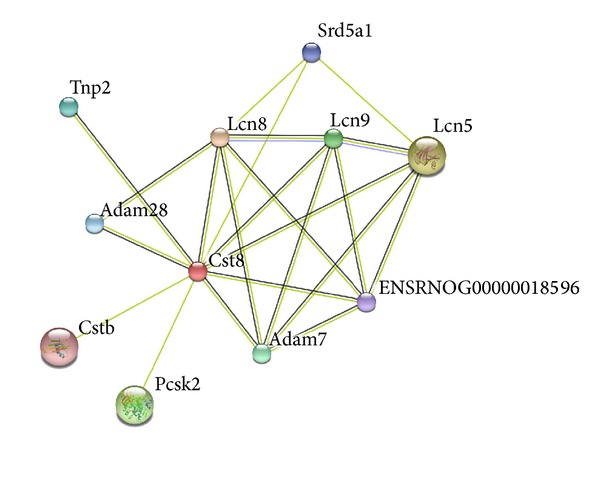
The PPI network of* Cst8 *gene.

In addition, the protein-protein interaction (PPI) network of* Rest* was constructed via STRING tool and displayed in Figure 3, suggesting that Rest protein might interact with 11 proteins in rats. The PPI network of* Cst8* was built as well, in which* Cst8 *was the hub protein connected with 10 proteins (Figure 4).

### 3.3. Enrichment Analysis of DEGs

For function analysis, all DEGs were inputted into DAVID for GO function and KEGG pathway enrichment analysis. *P* < 0.01 was set as significant difference.

GO function enrichment analysis of DEGs in earlier stage showed 170 significant GO terms, which were divided into 20 clusters, including material transportation in cells, regulation of biological process, structure development, neurodevelopment, and the blood pressure regulation. The most significant GO term was GO: 0051179 (localization), of which the fold enrichment was 1.576. The top 10 GO terms were shown in Table 3 (upper). Similarly, total 111 significant GO terms were obtained from GO analysis of DEGs in later stage and were divided into 13 clusters, including neurons and synapses development, ion transport, regulation of gene expression, and hormone secretion. The most significant GO term was GO: 0045202 (synapse), of which the fold enrichment was 2.53. The top 10 GO terms of later stage were shown in Table 3 (lower).

Additionally, KEGG pathway enrichment analysis of DEGs in earlier stage showed 5 significant pathways (Table 4, upper). Calcium signaling pathway was the most significant one (fold enrichment: 2.69). Meanwhile, DEGs in later stage were enriched into 2 significant pathways, mainly focused on neurodevelopment (Table 4, lower).

### 3.4. Correlation Analysis

Among the expressed genes after fracture, a Pearson correlation coefficient was calculated between gene expression level and fracture time via cor.test in R language. With the strict cut-off of *P* < 0.05, total 3,300 genes significantly associated with fracture time were collected, including negative correlation (1,714 genes) and positive correlation (1,586 genes) (Table 5). None of the 3,300 significant correlation genes was overlapped with DEGs identified using LIMMA package. The function annotation of these significant correlation genes showed relationship with illness, cancer, and immune system, indicating that surgical approach did not cause serious damage to health of animals. Besides, the correlation analysis of DEGs from two stages did not show significant correlation with fracture time.

## 4. Discussion

In daily life, sport-related fractures are common in adolescents, particularly in males [23]. Femoral fracture, a common sport injury, has great impact on human physical exercise ability and improper treatment can lead to nerve injury, infection, pain, or dyskinesia [5]. For professional athletes, femoral fracture is very popular and the outcome of treatment affects their athletic career [24]. It is necessary for us to identify DEGs after femoral fracture and to explore the key gene of the bone healing, which will provide theoretical basis for future treatment of these sport-related fractures.

In this study, the chip data were divided into earlier stage and later stage based on 7 time points after femoral fracture. In earlier and later stages, 1,004 and 986 DEGs were identified by comparing with control group, respectively. For example, among DEGs in early stage,* Oprm1 *was opioid receptor [25], the reduced expression of which in dorsal root ganglion neurons was found to be associated with bone cancer pain in mouse models [26].* Tmem200a *was a transmembrane protein which might inhibit overgrowth of myelocyte [27]. Meanwhile, among DEGs in later stage,* Bcl2l 1 *encoded Bcl-2-like 1 protein, a critical regulator of programmed cell death, belongs to Bcl-2 protein family [28]. Consistently, it is reported that Bcl-2 plays an important role in regulating the apoptosis of osteoclast and osteocyte [29]. Furthermore,* Wt1* (Wilms tumor 1) might act as a novel oncogene facilitating development of the lethal metastatic phenotype in prostate cancer [30].

Among DEGs between two stages, there was no significant difference in the number of DEGs and total 87 DEGs were shared by two stages, indicating different expression profiles between two stages. There were 4 DEGs oppositely regulated in earlier and later stages, which might act as indicators for femur healing. Among the 4 DEGs,* Rest*, similar to* Tmem200a*, might inhibit overgrowth of myelocyte combined with myc gene [31].* Rest *gene is a transcriptional repressor of diverse neuronal genes, the downregulation of which contributed to the proper development of neurons [32]. Similarly, in the current study,* Rest *was downregulated in earlier stage but upregulated in later stage. Moreover,* Rest* is involved in the differentiation from pluripotent cell to neural stem cell and from stem cell to mature neurons [33].* Cyst8* belongs to cystatin family of proteins [34]. Many members of the cystatin superfamily such as gelatin could protect matrix metalloproteinases without affecting their biological activities, which are critical for tissue modeling [35]. Total 57 DEGs were downregulated in both two stages, of which interaction network showed that 5 genes were interacted with other reported genes in rats, such as syt and stx families. Syt1 was a key factor controlling neurotransmitters release via binding to calcium ion [36]. Consistently, this study showed that calcium signaling pathway was also enriched in early stage, suggesting the critical role of calcium signaling in bone healing after femoral fracture. Besides, Syt1 might control neural signal transmission combined with SNAP-25 [37, 38] and STX1A [39]. STX1A was involved in vesicle fusion process which is critical for calcium-dependent neurotransmitters release. Importantly, it has been reported that increase of Syt-1 might play a role in impairment of learning and memories attributed to aging in mouse model [40].

Pearson's correlation coefficient analysis between gene expression and fracture time indicated that significant correlation genes between gene expression and fracture time were not overlapped with identified DEGs, which demonstrated that rats underwent surgical operation without other infections and injuries.

GO analysis of DEGs from two stages was enriched into different GO terms. Briefly, in earlier stage, abundant DEGs were related to material transportation and synthesis in cells, and a few genes were enriched in synapse growth, while, in later stage, in contrast, the majority of DEGs were related to synapse growth and a small number of genes were related to transporter activity. These discrepancies suggested that fracture healing involved distinct functions in earlier and later stages. Besides, system development was enriched in both earlier and later stages, revealing its importance in the whole process of fracture healing. KEGG pathway analysis showed that neuroactive ligand-receptor interaction was needed in two stages, indicating its important role in fracture healing. Meanwhile, in earlier stage, DEGs were significantly enriched into calcium signaling pathway and neuroactive ligand-receptor interaction pathway. For later stage, neuroactive ligand-receptor interaction becomes the most important pathway, and axon guidance pathway was also enriched. The two pathways were closely associated with neurodevelopment. These findings indicated difference of physical growth between two stages. In earlier stage, more nutrients for vegetative growth were needed to repair fracture; in later stage, nervous systems were repaired to restore movement ability, which were consistent with general understanding.

## 5. Conclusions

In conclusion, based on rat model, identification of DEGs after femoral fractures was useful for investigation of the proper treatment and providing foundation for exercise capacity recovery. However, further genetic studies were still needed to confirm our observation.

## Figures and Tables

**Table 1:** The most significant upregulated and downregulated DEGs (top 10 of each) from earlier stage.

Gene symbol	Full name	*P* value	log_2_⁡FC
Tmem200a	Transmembrane protein 200A	0.0000116	3.28
Oprm1	Opioid receptor, mu 1	0.0000297	3.31
Ccl20	Chemokine (C-C motif) ligand 20	0.0001817	1.17
Zbtb39	Zinc finger and BTB domain containing 39	0.0002876	2.32
LOC100910826	Uncharacterized LOC100910826	0.0002924	1.65
Rilpl1	Rab interacting lysosomal protein-like 1	0.0003576	1.57
Pemt	Phosphatidylethanolamine N-methyltransferase	0.0005798	2.17
Zc2hc1a	Zinc finger, C2HC-type containing 1A	0.0006232	2.38
Cdrt4	CMT1A duplicated region transcript 4	0.0009013	2.41
Ret	Ret proto-oncogene	0.0013837	1.13
Zfp278	Zinc finger protein 278	0.0000801	−2.24
Kiaa0415	KIAA0415 protein	0.0001213	−2.25
Nfib	Nuclear factor I/B	0.0001406	−2.33
Sycn	Syncollin	0.0002121	−1.9
Htr7	5-Hydroxytryptamine (serotonin) receptor 7	0.0002318	−2.05
Mpp2	Membrane protein, palmitoylated 2 (MAGUK p55 subfamily member 2)	0.0002686	−1.83
Scai	Suppressor of cancer cell invasion	0.000331	−2.09
Apoe	Apolipoprotein E	0.0004889	−2.49
Hrh1	Histamine receptor H 1	0.0005174	−2.41
Kiss1r	KISS1 receptor	0.0005912	−1.92

**Table 2:** The most significant upregulated and downregulated DEGs (top 10 of each) from later stage.

Gene symbol	Full name	*P* value	log_2_⁡FC
Bcl2l1	Bcl2-like 1	0.000093	2.37
Tenm2	Teneurin transmembrane protein 2	0.0002237	2.07
Chrm4	Cholinergic receptor, muscarinic 4	0.0003062	2.26
Kcnk10	Potassium channel, subfamily K, member 10	0.0004325	2.12
Tti2	TELO2 interacting protein 2	0.0006392	2.33
Spatc1	Spermatogenesis and centriole associated 1	0.0006546	2.91
Sulf1	Sulfatase 1	0.0009184	2.17
Ankrd55	Ankyrin repeat domain 55	0.0011436	2.05
Cacng8	Calcium channel, voltage-dependent, gamma subunit 8	0.001285	1.68
Drd1a	Dopamine receptor D1A	0.0013418	2.49
Ephx4	Epoxide hydrolase 4	0.0000341	−2.22
Wt1	Wilms tumor 1	0.0001725	−2.45
Trpv6	Transient receptor potential cation channel, subfamily V, member 6	0.0002677	−2.48
Shisa3	Shisa homolog 3 (*Xenopuslaevis*)	0.000375	−3.05
Spink8	Serine peptidase inhibitor, Kazal type 8	0.0004495	−2.13
Ank1	Ankyrin 1, erythrocytic	0.0007826	−1.45
Acsbg1	Acyl-CoA synthetase bubblegum family member 1	0.0009768	−2.58
Rcbtb2	Regulator of chromosome condensation (RCC1) and BTB (POZ) domain containing protein 2	0.0012021	−1.92
Ninj2	Ninjurin 2	0.001204	−2.08
Nog	Noggin	0.0016676	−2.58

**Table 3:** GO enrichment analysis of DEGs in earlier stage (upper) and later stage (lower).

Category	Term	Gene number	*P* value	Fold enrichment
Earlier stage				
GOTERM_BP_ALL	GO:0051179~localization	140	5.56*E* − 09	1.57
GOTERM_BP_ALL	GO:0048731~system development	121	9.53*E* − 09	1.64
GOTERM_BP_ALL	GO:0051234~establishment of localization	122	3.92*E* − 08	1.60
GOTERM_BP_ALL	GO:0065007~biological regulation	249	4.01*E* − 08	1.29
GOTERM_CC_ALL	GO:0045202~synapse	38	4.14*E* − 08	2.76
GOTERM_BP_ALL	GO:0006810~transport	121	4.16*E* − 08	1.60
GOTERM_BP_ALL	GO:0032502~developmental process	141	4.71*E* − 08	1.52
GOTERM_BP_ALL	GO:0007275~multicellular organismal development	129	1.15*E* − 07	1.54
GOTERM_BP_ALL	GO:0048856~anatomical structure development	122	1.27*E* − 07	1.57
GOTERM_BP_ALL	GO:0048666~neuron development	34	2.41*E* − 07	2.76
Later stage				
GOTERM_CC_ALL	GO:0045202~synapse	32	3.67*E* − 06	2.53
GOTERM_BP_ALL	GO:0051179~localization	122	4.65*E* − 06	1.46
GOTERM_MF_ALL	GO:0022838~substrate specific channel activity	30	5.34*E* − 06	2.59
GOTERM_CC_ALL	GO:0044456~synapse part	25	5.88*E* − 06	2.90
GOTERM_MF_ALL	GO:0022803~passive transmembrane transporter activity	30	1.08*E* − 05	2.50
GOTERM_MF_ALL	GO:0015267~channel activity	30	1.08*E* − 05	2.50
GOTERM_BP_ALL	GO:0048731~system development	102	2.89*E* − 05	1.47
GOTERM_MF_ALL	GO:0005215~transporter activity	61	3.17*E* − 05	1.73
GOTERM_MF_ALL	GO:0005261~cation channel activity	23	3.29*E* − 05	2.76
GOTERM_BP_ALL	GO:0030001~metal ion transport	31	3.31*E* − 05	2.30

**Table 4:** KEGG pathway analyses of DEGs in earlier stage (upper) and later stage (lower).

Category	Term	Gene number	*P* value	Fold enrichment
Earlier stage				
KEGG_PATHWAY	rno04020: calcium signaling pathway	18	3.09*E* − 04	2.70
KEGG_PATHWAY	rno00980: metabolism of xenobiotics by cytochrome P450	9	0.001370145	4.09
KEGG_PATHWAY	rno04080: neuroactive ligand-receptor interaction	20	0.003048821	2.08
KEGG_PATHWAY	rno00982: drug metabolism	9	0.004411985	3.41
KEGG_PATHWAY	rno02010: abc transporters	7	0.004863078	4.34
Later stage				
KEGG_PATHWAY	rno04080: neuroactive ligand-receptor interaction	23	6.18*E* − 05	2.58
KEGG_PATHWAY	rno04360: axon guidance	12	0.003586	2.78

**Table 5:** The most significant negative and positive correlation between gene expression level and fracture time at *P* value < 0.005 (top 10 of each).

GenBankAcc	Coefficient	*P* value
AA859496	−0.99368	6.08*E* − 06
AI406518	−0.99112	1.42*E* − 05
AA892299	−0.99081	1.55*E* − 05
AW524669	−0.98737	3.42*E* − 05
BE104302	−0.98477	5.45*E* − 05
AW532414	−0.98284	7.34*E* − 05
BM386669	−0.97924	0.000118
BE115521	−0.97904	0.000121
NM_019243	−0.97884	0.000124
BM383832	−0.97759	0.000143
BF411794	0.990571	1.65*E* − 05
AI412189	0.989311	2.26*E* − 05
BG669998	0.989281	2.27*E* − 05
BF412924	0.981708	8.61*E* − 05
BF399367	0.981053	9.39*E* − 05
BE098337	0.979558	0.000113
AA943135	0.97682	0.000155
BF284937	0.976587	0.000159
BI295973	0.973675	0.000213
AI236953	0.970683	0.000278

GenBankAcc: GenBank accession number.

## References

[1] Rome ES (1995). Sports-related injuries among adolescents: when do they occur, and how can we prevent them?. *Pediatrics in Review*.

[2] Bennell KL, Brukner PD (1997). Epidemiology and site specificity of stress fractures. *Clinics in Sports Medicine*.

[3] Gilmour R Adjustable knee brace.

[4] Hedin H (2004). Surgical treatment of femoral fractures in children—comparison between external fixation and elastic intramedullary nails: a review. *Acta Orthopaedica Scandinavica*.

[5] Palmu S, Paukku R, Peltonen J, Nietosvaara Y (2010). Treatment injuries are rare in children's femoral fractures: compensation claims submitted to the Patient Insurance Center in Finland. *Acta Orthopaedica*.

[6] Reeves RB, Ballard RI, Hughes JL (1990). Internal fixation versus traction and casting of adolescent femoral shaft fractures. *Journal of Pediatric Orthopaedics*.

[7] Kregor PJ, Stannard JA, Zlowodzki M, Cole PA (2004). Treatment of distal femur fractures using the less invasive stabilization system: surgical experience and early clinical results in 103 fractures. *Journal of Orthopaedic Trauma*.

[8] Apivatthakakul T, Chiewcharntanakit S (2009). Minimally invasive plate osteosynthesis (MIPO) in the treatment of the femoral shaft fracture where intramedullary nailing is not indicated. *International Orthopaedics*.

[9] Dousa P, Bartonicek J, Lunacek L, Pavelka T, Kusikova E (2011). Ipsilateral fractures of the femoral neck, shaft and distal end: long-term outcome of five cases. *International Orthopaedics*.

[10] Barei DP, Schildhauer TA, Nork SE (2003). Noncontiguous fractures of the femoral neck, femoral shaft, and distal femur. *The Journal of Trauma*.

[11] Bollen AM, Bai XQ (2005). Effects of long-term calcium intake on body weight, body fat and bone in growing rats. *Osteoporosis International*.

[12] Meyer RA, Meyer MH, Ashraf N, Frick S (2007). Changes in mRNA gene expression during growth in the femoral head of the young rat. *Bone*.

[13] Ashraf N, Meyer MH, Frick S, Meyer RA (2007). Evidence for overgrowth after midfemoral fracture via increased RNA for mitosis. *Clinical Orthopaedics and Related Research*.

[14] Irizarry RA, Hobbs B, Collin F (2003). Exploration, normalization, and summaries of high density oligonucleotide array probe level data. *Biostatistics*.

[15] Smyth GK (2005). Limma: linear models for microarray data. *Bioinformatics and Computational Biology Solutions Using R and Bioconductor*.

[16] Jensen LJ, Kuhn M, Stark M (2009). STRING 8—s global view on proteins and their functional interactions in 630 organisms. *Nucleic Acids Research*.

[17] Martin A, Ochagavia ME, Rabasa LC, Miranda J, Fernandez-de-Cossio J, Bringas R (2010). BisoGenet: a new tool for gene network building, visualization and analysis. *BMC Bioinformatics*.

[18] Huang DW, Sherman BT, Lempicki RA (2008). Systematic and integrative analysis of large gene lists using DAVID bioinformatics resources. *Nature Protocols*.

[19] Huang-da W, Sherman BT, Lempicki RA (2009). Bioinformatics enrichment tools: paths toward the comprehensive functional analysis of large gene lists. *Nucleic Acids Research*.

[20] Kanehisa M (2002). The KEGG database. *Novartis Foundation Symposium*.

[21] Hulsegge I, Kommadath A, Smits MA (2009). Globaltest and GOEAST: two different approaches for Gene Ontology analysis. *BMC Proceedings*.

[22] Langfelder P, Horvath S (2012). Fast R functions for robust correlations and hierarchical clustering. *Journal of Statistical Software*.

[23] Goulding A (2007). Risk factors for fractures in normally active children and adolescents. *Medicine and Sport Science*.

[24] Matheson GO, Clement DB, Mckenzie DC, Taunton JE, Lloyd-Smith DR, MacIntyre JG (1987). Stress fractures in athletes. A study of 320 cases. *The American Journal of Sports Medicine*.

[25] Zhang Y, Wang D, Johnson AD, Papp AC, Sadée W (2005). Allelic expression imbalance of human mu opioid receptor (OPRM1) caused by variant A118G. *The Journal of Biological Chemistry*.

[26] Yamamoto J, Kawamata T, Niiyama Y, Omote K, Namiki A (2008). Down-regulation of mu opioid receptor expression within distinct subpopulations of dorsal root ganglion neurons in a murine model of bone cancer pain. *Neuroscience*.

[27] Hui ABY, Takano H, Lo KW (2005). Identification of a novel homozygous deletion region at 6q23.1 in medulloblastomas using high-resolution array: comparative genomic hybridization analysis. *Clinical Cancer Research*.

[28] Beverly LJ, Howell LA, Hernandez-Corbacho M, Casson L, Chipuk JE, Siskind LJ (2013). BAK activation is necessary and sufficient to drive ceramide synthase-dependent ceramide accumulation following inhibition of BCL2-like proteins. *Biochemical Journal*.

[29] Wiren KM, Toombs AR, Semirale AA, Zhang X (2006). Osteoblast and osteocyte apoptosis associated with androgen action in bone: requirement of increased Bax/Bcl-2 ratio. *Bone*.

[30] Brett A, Pandey S, Fraizer G (2013). The Wilms’ tumor gene (WT1) regulates E-cadherin expression and migration of prostate cancer cells. *Prostate*.

[31] Su X, Gopalakrishnan V, Stearns D (2006). Abnormal expression of REST/NRSF and Myc in neural stem/progenitor cells causes cerebellar tumors by blocking neuronal differentiation. *Molecular and Cellular Biology*.

[32] Paquette AJ, Perez SE, Anderson DJ (2000). Constitutive expression of the neuron-restrictive silencer factor (NRSF)/REST in differentiating neurons disrupts neuronal gene expression and causes axon pathfinding errors in vivo. *Proceedings of the National Academy of Sciences of the United States of America*.

[33] Ballas N, Grunseich C, Lu DD, Speh JC, Mandel G (2005). REST and its corepressors mediate plasticity of neuronal gene chromatin throughout neurogenesis. *Cell*.

[34] Ochieng J, Chaudhuri G (2010). Cystatin superfamily. *Journal of Health Care for the Poor and Underserved*.

[35] Ray S, Lukyanov P, Ochieng J (2003). Members of the cystatin superfamily interact with MMP-9 and protect it from autolytic degradation without affecting its gelatinolytic activities. *Biochimica et Biophysica Acta—Proteins and Proteomics*.

[36] Lee H-K, Yang Y, Su Z (2010). Dynamic Ca^2+^-dependent stimulation of vesicle fusion by membrane-anchored synaptotagmin 1. *Science*.

[37] Gerona RRL, Larsen EC, Kowalchyk JA, Martin TFJ (2000). The C terminus of SNAP25 is essential for Ca^2+^-dependent binding of synaptotagmin to SNARE complexes. *Journal of Biological Chemistry*.

[38] Lynch KL, Gerona RRL, Larsen EC, Marcia RF, Mitchell JC, Martin TFJ (2007). Synaptotagmin C2A Loop 2 mediates Ca2+-dependent SNARE interactions essential for Ca2+-triggered vesicle exocytosis. *Molecular Biology of the Cell*.

[39] Shao X, Li C, Fernandez I, Zhang X, Südhof TC, Rizo J (1997). Synaptotagmin-syntaxin interaction: the C_2_ domain as a Ca^2+^-dependent electrostatic switch. *Neuron*.

[40] Chen GH, Wang YJ, Qin S, Yang QG, Zhou JN, Liu RY (2007). Age-related spatial cognitive impairment is correlated with increase of synaptotagmin 1 in dorsal hippocampus in SAMP8 mice. *Neurobiology of Aging*.

